# Device embolization into the LV following left atrial endage closure with an Amplatzer Amulet

**DOI:** 10.1007/s10840-018-0374-7

**Published:** 2018-05-03

**Authors:** Krzysztof Kaczmarek, Bartosz Czarniak, Piotr Jakubowski, Pawel Ptaszynski

**Affiliations:** 1 Department of Electrocardiology, Medical University of Lodz, Lodz, Poland; 2 Department of Cardiology, Medical University of Lodz, Lodz, Poland

A 74-year-old man with paroxysmal atrial fibrillation, severe ischemic heart failure, after surgical coronary artery revascularization and implantation of cardiac resynchronization cardioverter-defibrillator was referred for left atrial appendage closure (LAAC). He was treated with dabigatran because of INR lability on warfarin. Nevertheless, the patient suffered acute life-threatening gastrointestinal bleeding a few months ago. The procedure of LAAC was performed under local anesthesia via the right femoral vein and with transoesophageal echocardiography (TOE) guidance. After transseptal puncture, a cardioangiography revealed a cone-shaped windsock left atrial appendage (LAA). The landing zone measured on fluoroscopy was 21 mm. A 25-mm Amplatzer Amulet device was deployed; however, it failed the stability tests. Therefore, remeasurements were performed, and finally, a 28-mm plug was chosen and successfully deployed without any leak. Despite a good outcome in the stability test, little compression of the lobe was observed (Fig. 1). The deployment of the occluder was suboptimal but was deemed as the best possible. Nonetheless, a routine transthoracic echocardiography the next day revealed embolization of the device into the apical area of the LV (Fig. 2), without any impairment of mitral valve function. The patient was asymptomatic and remained clinically stable. LAA occluder embolization is a rare but known complication and can be managed both surgically and percutaneously [1]. Under local anesthesia, common femoral artery access was obtained with an 80-cm 16F Check-Flo Performer Introducer (Cook Medical Inc.). A 7F JR 4.0 guiding catheter was placed through a sheath into the left ventricle, and a 30-mm Multisnare device (Braun Interventional Systems Inc.) was introduced to snare the occluder. Subsequently, the device was carefully moved into the left ventricular outflow tract and pulled across the aortic valve to the ascending aorta. The recapturing maneuver was performed in the abdominal aorta. TOE ruled out any mechanical damage to the aortic valve. No complication was observed in the 6 months follow-up. We obtained the following experiences from our procedure. There were three critical moments which allowed the procedure to be performed successfully and without complications. The first difficulty was to grab the device in the left ventricle. Secondly, the device was brought to the ascending aorta through the aortic valve. A firm and continuous pullback got the occluder stuck in the aortic valve and subsequently made it jump into the aorta (Movies 2 and 3). Finally, to prevent any cerebral events, the occluder was pulled back into the sheath in the descending aorta. A challenging anatomy of the LAA and a sinus rhythm are the principal risk factors of device embolization [2, 3]. However, some authors hypothesize that device embolization can occur without obvious explanation, and it can be associated with limitations of the method. In our case, prior to device release, four signs of proper device placement were observed. The distal part of the lobe was slightly over-compressed. Therefore, adhesion of the proximal part of the lobe to the LAA wall was incomplete, and this could be the cause of embolization The anatomy of the LAA might have been unfavorable for the Amplatzer Amulet device [3]. The patient presented in this manuscript is one of the few examples supporting the percutaneous approach for the retrieval of embolized LAA occluders as a safe and efficacious solution for such complication.

**Fig. 1:**
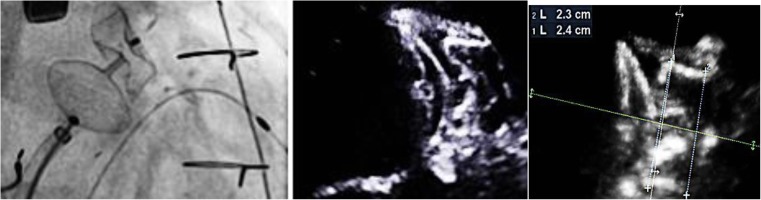
A successful deployment of a 28-mm Amulet with TOE measurements

**Fig. 2:**
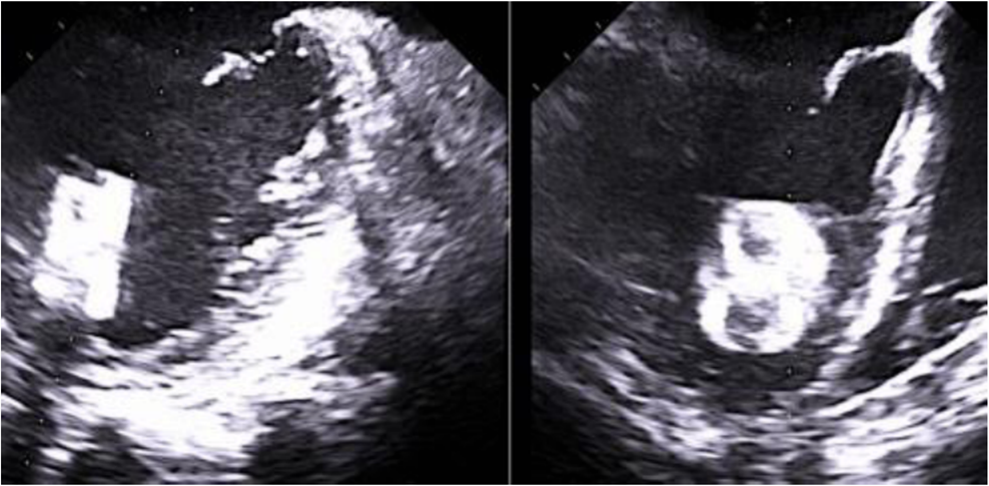
Device embolization into the LV in TOE

## Electronic supplementary material

ESM 1 (MP4 4772 kb)

ESM 2 (MP4 942 kb)

ESM 3 (MP4 2326 kb)
